# Low nuclear body formation and tax SUMOylation do not prevent NF-kappaB promoter activation

**DOI:** 10.1186/1742-4690-9-77

**Published:** 2012-09-25

**Authors:** Amandine Bonnet, Voahangy Randrianarison-Huetz, Patrycja Nzounza, Martine Nedelec, Maxime Chazal, Laetitia Waast, Sabrina Pene, Ali Bazarbachi, Renaud Mahieux, Laurence Bénit, Claudine Pique

**Affiliations:** 1 INSERM, U1016, Institut Cochin, 22 rue Méchain, 75014 Paris, France; 2 CNRS, UMR8104, Paris, France; 3 Université Paris Descartes, Sorbonne Paris Cité, Paris, France; 4 Department of Internal Medicine, American University of Beirut, Beirut, Lebanon; 5 INSERM, U758, Ecole Normale Supérieure de Lyon, Lyon, France

**Keywords:** Retrovirus, Leukemia, NF-kappaB, Ubiquitin, SUMO, Nuclear speckles

## Abstract

**Background:**

The Tax protein encoded by Human T-lymphotropic virus type 1 (HTLV-1) is a powerful activator of the NF-κB pathway, a property critical for HTLV-1-induced immortalization of CD4^+^ T lymphocytes. Tax permanently stimulates this pathway at a cytoplasmic level by activating the IκB kinase (IKK) complex and at a nuclear level by enhancing the binding of the NF-κB factor RelA to its cognate promoters and by forming nuclear bodies, believed to represent transcriptionally active structures. In previous studies, we reported that Tax ubiquitination and SUMOylation play a critical role in Tax localization and NF-κB activation. Indeed, analysis of lysine Tax mutants fused or not to ubiquitin or SUMO led us to propose a two-step model in which Tax ubiquitination first intervenes to activate IKK while Tax SUMOylation is subsequently required for promoter activation within Tax nuclear bodies. However, recent studies showing that ubiquitin or SUMO can modulate Tax activities in either the nucleus or the cytoplasm and that SUMOylated Tax can serve as substrate for ubiquitination suggested that Tax ubiquitination and SUMOylation may mediate redundant rather than successive functions.

**Results:**

In this study, we analyzed the properties of a new Tax mutant that is properly ubiquitinated, but defective for both nuclear body formation and SUMOylation. We report that reducing Tax SUMOylation and nuclear body formation do not alter the ability of Tax to activate IKK, induce RelA nuclear translocation, and trigger gene expression from a NF-κB promoter. Importantly, potent NF-κB promoter activation by Tax despite low SUMOylation and nuclear body formation is also observed in T cells, including CD4^+^ primary T lymphocytes. Moreover, we show that Tax nuclear bodies are hardly observed in HTLV-1-infected T cells. Finally, we provide direct evidence that the degree of NF-κB activation by Tax correlates with the level of Tax ubiquitination, but not SUMOylation.

**Conclusions:**

These data reveal that the formation of Tax nuclear bodies, previously associated to transcriptional activities in Tax-transfected cells, is dispensable for NF-κB promoter activation, notably in CD4^+^ T cells. They also provide the first evidence that Tax SUMOylation is not a key determinant for Tax-induced NF-κB activation.

## Background

Human T-lymphotropic virus type 1 (HTLV-1) is the agent of Adult T-cell Leukemia, a fatal hematopoietic malignancy due to the transformation of CD4^+^ T lymphocytes. The Tax regulatory viral protein plays a pivotal role in HTLV-1-induced T-cell transformation. Indeed, Tax triggers permanent T cell proliferation through a variety of mechanisms including promotion of cell cycle, deregulation of apoptosis and activation or repression of cellular gene promoters (reviewed in [1-4]). Notably, Tax is a powerful inducer of the NF-κB pathway, an activity shown to be required for HTLV-1-induced immortalization of primary CD4^+^ T lymphocytes [5].

In physiological conditions, the NF-κB pathway is transiently activated in response to extracellular stimuli (reviewed in [6]). These results in the activation of the cytoplasmic IκB kinase (IKK) complex, which consists of two catalytic subunits, IKKα and IKKβ and a regulatory subunit, NF-κB essential modulator (NEMO)/IKKγ. Once activated, IKK induces the phosphorylation and degradation of the NF-κB inhibitors IκB, liberating the NF-κB factors which then translocate to the nucleus. Contrasting with the physiological situation, Tax is able to activate the NF-κB pathway in a permanent manner by acting on both the cytoplasmic and nuclear phases. In the cytoplasm, Tax binds to NEMO [7] and recruits adaptor proteins and kinases that in turn promote IKKα/β activation ([8-11] and reviewed in [12]). In the nucleus, Tax binds to and stabilizes the binding of NF-κB factors, including RelA/p65, to the NF-κB dependent promoter [13,14]. In the nucleus, Tax also assembles into particular structures called Tax nuclear speckled structures or Tax nuclear bodies [15-17]. This was shown to depend upon the presence of an N-terminal region located between residues 50 to 75 of Tax (Tax speckled structure localization signal, TSLS) [18]. NF-κB-mediated transcription may arise in these structures, since they contain components of the NF-κB pathway such as p50, RelA/p65 and NEMO [16,19,20]. In addition, repression of cellular promoters was also associated with Tax nuclear bodies [21]. Tax nuclear bodies also contain components of splicing complexes [17] and DNA damage response machineries [22] and were, therefore, proposed to mediate other functions than transcription (reviewed in [23]).

In previous studies, we and others demonstrated that Tax is ubiquitinated and SUMOylated [20,24-26]. A series of studies focusing on the role of Tax ubiquitination demonstrated the critical role of this modification in NF-κB activation. Indeed, reducing or increasing Tax ubiquitination by interfering with ubiquitination or deubiquitination enzymes was shown to block or enhance Tax-induced NF-κB activation, respectively [27-30]. NF-κB activation was associated with Tax conjugation to K63-linked ubiquitin chains, which were shown to be essential for Tax binding to NEMO and IKK activation [30-32]. K63-linked ubiquitin chains also promote the targeting of Tax and NEMO to perinuclear spots associated to the centrosome and the Golgi apparatus, believed to represent a Tax-induced cytoplasmic signaling platform [32,33]. Tax SUMOylation was initially associated with nuclear events. Indeed, SUMO-1-conjugated Tax subpopulations were found in the nucleus and coexpressing Tax along with SUMO-1 was shown to increase the nuclear fraction of Tax. Moreover, SUMO-1 was found to colocalize with Tax in nuclear bodies [19,20,24].

**Figure 1:**
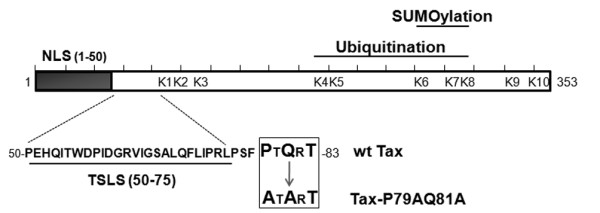
**Description of the Tax-P79AQ81A mutant.** Schematic representation of the primary amino-acid sequence of Tax showing the N-terminal nuclear localization signal (NLS, amino-acids 1–50), the Tax speckled structure localization signal (TSLS, amino-acids 50–75) and the potential TRAF-binding motif (boxed) in which alanine substitutions were introduced (Tax-P79AQ81A mutant). The lysine residues targeted by ubiquitination and SUMOylation are also indicated.

Tax possesses 10 lysine residues (referred to as K1 to K10), among those K4 to K8 serve as targets for ubiquitination and K6 to K8 as targets for SUMOylation (Figure1). We and others previously showed that mutating lysines K4 to K8 abolishes both Tax ubiquitination and SUMOylation and renders Tax inactive for RelA nuclear translocation, a defect restored by making a Tax ubiquitin fusion protein. Mutating only lysines among K6 to K8 still allows RelA nuclear translocation, but strongly reduces NF-κB promoter activation, which is partially restored upon fusion to SUMO-1 [20,24]. These findings led to the proposition of a two-step working model in which K63 ubiquitination of Tax first intervenes in the cytoplasm to activate IKK and allows RelA nuclear translocation, while Tax SUMOylation is subsequently required for RelA-dependent promoter activation within Tax nuclear bodies. However, subsequent observations suggest a more complex picture. Indeed, it was reported that mono-ubiquitination in the nucleus activates the nucleocytoplasmic shuttling of Tax in stress conditions [34]. Moreover, fusing lysine Tax mutants to SUMO-1 was shown to enhance NEMO targeting to cytoplasmic perinuclear spots while fusing them to ubiquitin was shown to restore the formation of nuclear bodies [19]. In addition, RNF4, an ubiquitin ligase that preferentially ubiquitinates SUMOylated substrates, was recently reported to be involved in Tax ubiquitination [27]. These recent findings favor the notion that Tax ubiquitination and SUMOylation may mediate redundant rather than successive functions. 

How Tax post-translational modifications and their impact on Tax localization are synchronized with NF-κB activation is still unclear. Moreover, the importance of Tax post-translational modifications and nuclear bodies has not been investigated in CD4^+^ T cells yet. In this study, we revisited the role of Tax nuclear bodies and Tax SUMOylation on Tax activities. We confirm that Tax SUMOylation correlates with the formation of Tax nuclear bodies. We also demonstrate that, surprisingly, impaired nuclear body formation still allows Tax to fully activate a NF-κB promoter in either cell lines or primary CD4^+^ T cells. In addition, we provide evidence that the degree of Tax NF-κB activity correlates with the level of Tax ubiquitination but not SUMOylation. These data provide the first direct evidence that Tax nuclear body formation and Tax SUMOylation are dispensable for Tax-induced NF-κB activation.

## Results

### Tax-P79AQ81A, a Tax mutant defective for nuclear body formation

In the search for potential functional motifs in Tax, we selected a PxQxT sequence (aa 79–83) because this sequence fits with a putative motif for binding to the TRAFs (TNF-receptor associated factor), which are ubiquitin ligases acting in the NF-κB pathway [35]. Interestingly, the PxQxT motif is also just adjacent to the TSLS (Figure1), suggesting that it might be involved in the nucleocytoplasmic trafficking of Tax. It was previously shown that residues at position 1 and 3 of the PxQxT motif are the most critical for binding to the TRAFs [36]. Hence, a mutant in which the P79 and Q81 residues were changed to alanines (Tax-P79AQ81A, Figure1) was generated to study the role of the PxQxT motif in Tax modifications and activities.

**Figure 2:**
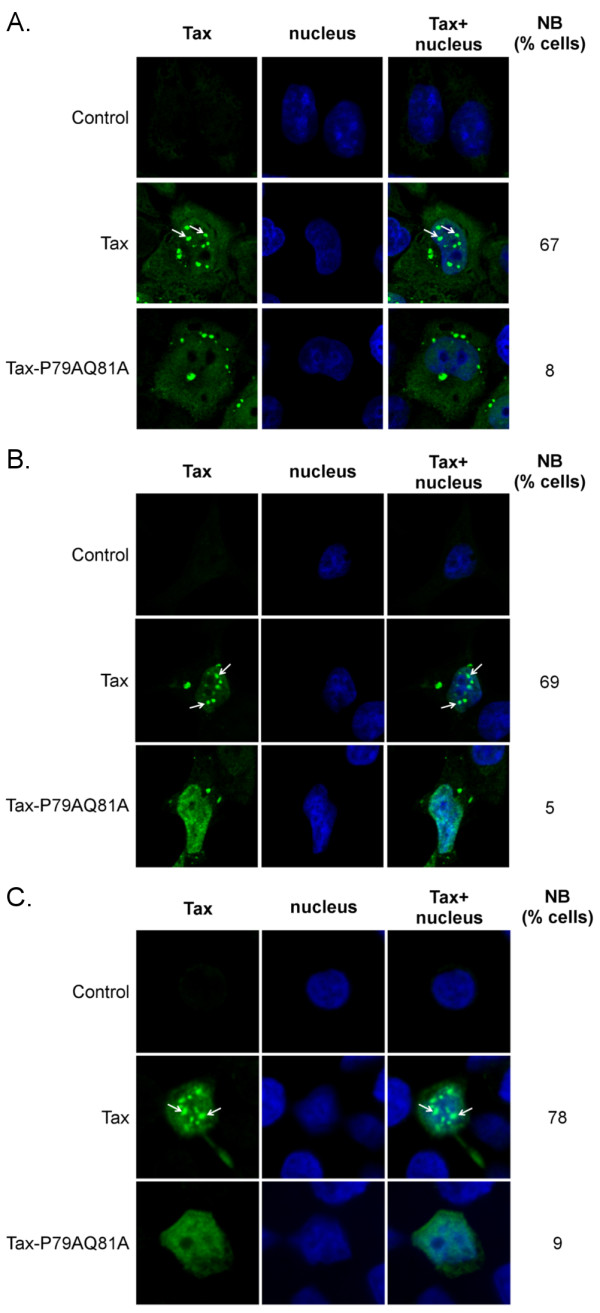
**Tax-P79AQ81A is defective for nuclear body formation.** Confocal microscopy analysis performed in (**A**) HeLa cells; (**B**) 293 T cells or (**C**) CEM T cells showing the distribution of wt Tax or Tax-P79AQ81A (green). Nuclei were stained with DAPI (blue). The percentages of cells containing Tax in nuclear bodies (NB) are indicated, and Tax nuclear bodies are pointed with arrows. At least 100 cells were analyzed in each condition.

The subcellular localization of wild-type (wt) Tax and Tax-P79AQ81A was first compared in HeLa cells (Figure2A). As previously described [24], wt Tax was detected both in the cytoplasm and in the nucleus, where it formed well visible nuclear bodies (NB) (67% of the cells). A dramatic change in nuclear localization was found for Tax-P79AQ81A since Tax-P79AQ81A was present in the nucleus as diffuse staining in all transfected cells, and very few and very small nuclear bodies were detected in only 8% of the cells while 92% of Tax positive cells did not show any nuclear bodies (Figure2A). A dramatic reduction in nuclear body formation by Tax-P79AQ81A was also found in transfected 293 T cells and more importantly, in transfected CEM T cells (less than 10% of the cells, Figure2B and 2C). 

Hence, mutations of the P79 and Q81 residues do not alter the nuclear import of Tax, but preclude Tax nuclear body formation. This confirms the importance of the TSLS-containing N-terminal region of Tax in nuclear body formation and suggests that the P79 and Q81 residues are part of this nuclear body targeting signal.

### Tax-P79AQ81A properly activates the cytoplasmic steps of the NF-κB pathway

**Figure 3:**
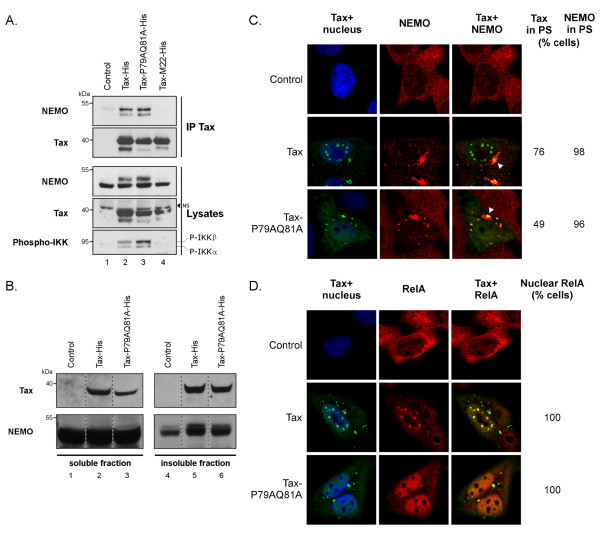
**Tax-P79AQ81A activates the cytoplasmic steps of the NF-**κ**B pathway.** (**A**) Ability of wt Tax and Tax-P79AQ81A to bind to endogenous NEMO and induce the phosphorylation of IKKα/β subunits. HeLa cells were transfected with a control plasmid or with the Tax-His constructs, including the NF-κB-defective Tax-M22 mutant as a negative control. Proteins precipitated using the anti-Tax mab (IP Tax) were blotted with either an anti-NEMO antibody or with the anti-Tax sera. In parallel, total proteins (Lysates) were blotted with anti-Tax sera and with anti-NEMO or anti-phospho-IKK antibodies, as indicated. NS: non specific. (**B**) Purification of aggresomes formed in wt Tax or Tax-P79AQ81A producing HeLa cells. Soluble and insoluble fractions were prepared according to [37] and proteins in each fraction were blotted with either an anti-NEMO antibody or the anti-Tax sera. (**CD**) Confocal microscopy analysis performed in HeLa cells showing the distribution of Tax (green) and endogenous NEMO (**C**) or RelA (**D**) (red). Nuclei were stained with DAPI (blue). At least 100 cells were analyzed in each condition.

In the cytoplasm, Tax binds to NEMO and activates the IKK complex, a process that requires the targeting of both Tax and IKK to perinuclear spots (PS) [32,33]. In immunoprecipitation assays, we confirmed that wt Tax coprecipitated endogenous NEMO and induced the phosphorylation of IKKα/β (Figure3A, lane 2). Tax-P79AQ81A also properly coprecipitated with endogenous NEMO and induced the phosphorylation of IKKα/β (Figure3A, lane 3). In contrast, and as expected from earlier data [7], neither NEMO binding nor IKK activation was observed for the NF-κB-defective M22 mutant (Figure3A, lane 4). 

A cell fractionation procedure described for the purification of cytoplasmic aggresomes, which are centrosomal-associated insoluble structures containing ubiquitinated proteins [37], was used next to study Tax and NEMO targeting to perinuclear spots. Comparable amounts of wt Tax and Tax-P79AQ81A were found in the insoluble fractions, indicating that both proteins were similarly targeted to cytoplasmic aggresomes (Figure3B, Tax panel, lanes 5 and 6). Similar enrichment of NEMO was observed in the insoluble fraction of cells expressing either wt Tax or Tax-P79AQ81A (Figure3B, NEMO panel, lanes 5 and 6), as compared to mock transfected cells (lane 4), indicating that both Tax proteins were equally efficient to relocalize NEMO to cytoplasmic aggresomes.

Confocal microscopy experiments further showed that endogenous NEMO was targeted to perinuclear spots in almost 100% of Tax-positive cells regardless of whether the cells had they produced wt Tax or Tax-P79AQ81A (Figure3C). Wt Tax was also concentrated in perinuclear spots in 76% of cells as compared to 49% of Tax-P79AQ81A-expressing cells (Figure3C). Of note, this difference was due to the reduction of the cell population containing Tax-P79AQ81A in both perinuclear spots and nuclear bodies while the proportion of cells containing Tax only in perinuclear spots was comparable between Tax-P79AQ81A and wt Tax (Additional file 1: Figure S1).

Since the final consequence of IKK activation is the nuclear translocation of RelA, we analyzed the localization of endogenous RelA in Tax-expressing cells (Figure3D). While RelA was found in the cytoplasm of Tax-negative cells, it was clearly relocalized to the nucleus in the totality of cells expressing either wt Tax or Tax-P79AQ81A. Interestingly, the pattern of RelA mirrored that of Tax since while RelA was found in nuclear bodies in cells producing wt Tax, it was detected as a diffuse staining in cells producing Tax-P79AQ81A (Figure3D).

These results demonstrate that Tax-P79AQ81A properly activates the cytoplasmic steps of the NF-κB pathway and induces nuclear RelA translocation in absence of nuclear body formation.

### Tax-P79AQ81A is as active as wt Tax for NF-κB promoter activation

**Figure 4:**
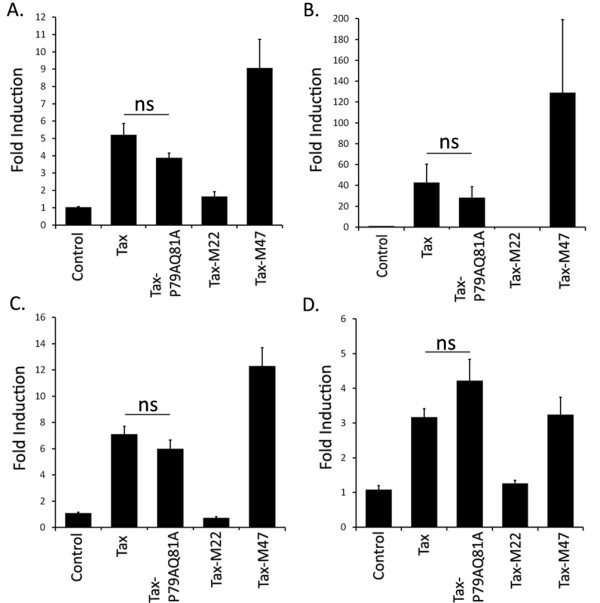
**Tax-P79AQ81A properly activates a NF-**κ**B promoter in either cell lines or primary CD4**^**+**^**T cells.** NF-κB promoter activity in Tax-transfected HeLa cells (**A**), 293 T cells (**B**), CEM T cells (**C**), and primary CD4^+^ T cells (**D**). Cells were transfected with a control plasmid or with the Tax-His constructs along with the NF-κB reporter plasmid and the Renilla luciferase expression plasmid for normalization. To validate the experiments, the M22 (defective for the NF-κB pathway) and M47 (defective for the CREB pathway) mutants were included in each experiment. Fold induction was calculated by dividing the firefly/renilla ratio of each Tax protein with the firefly/renilla ratio obtained with the control plasmid. The results represent the means and standard error of the means (SEM) from at least four independent experiments performed in duplicates. ns: not statistically significant.

Nuclear body formation was proposed to facilitate activation of the NF-κB pathway at the nuclear level. Whether Tax-P79AQ81A is able to drive gene expression from a NF-κB promoter was, therefore, investigated. NF-κB reporter gene assays performed in HeLa (Figure4A) and 293 T cells (Figure4B) showed no significant difference (p > 0.05) between the NF-κB promoter transactivation levels of wt Tax and Tax-P79AQ81A. The same experiments were performed in T cells, including the CEM T cell line (Figure4C) and primary CD4^+^ T cells (Figure4D). In both T cell systems, Tax-P79AQ81A was also fully able to transactivate the NF-κB promoter. Furthermore, Tax-P79AQ81A transactivated a CREB promoter similarly to wt Tax in all cell types, confirming that the protein was functional (Additional file 2: Figure S2A-D). In all reporter gene assays, Tax-M22, defective for NF-κB activation and Tax-M47, defective for CREB activation, were included as controls. Hence, while it is unable to form nuclear bodies, Tax-P79AQ81A is fully active in term of NF-κB promoter activation in both adherent cells and T cells, including primary CD4^+^ T cells.

### Tax nuclear bodies are hardly detected in HTLV-1-infected T cells

**Figure 5:**
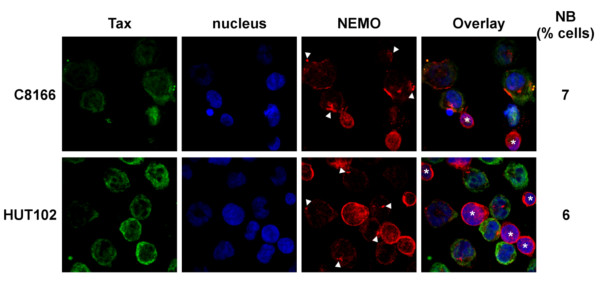
**Tax nuclear bodies are not detected in HTLV-1-infected T cell lines.** Confocal microscopy analysis performed with the HTLV-1-infected cell lines C8166 and HUT-102 showing the distribution of endogenous Tax (green) or NEMO (red). Nuclei were labeled with DAPI (blue). C8166 or HUT-102 cells were mixed with HTLV-1-negative CEM T cells as an internal negative control (indicated by asterisks). Perinuclear clusters of NEMO in Tax-positive cells are pointed with arrow heads, and the percentages of cells containing Tax in nuclear bodies (NB) are indicated. At least 200 cells were analyzed in each condition.

The previous findings, suggesting that absence of nuclear bodies did not alter Tax-induced NF-κB activation, prompted us to investigate the status of nuclear bodies in HTLV-1-infected T cells. Confocal microscopy experiments were performed in two HTLV-1-infected T cell lines, C8166 cells that contain defective HTLV-1 proviruses still allowing Tax production [38] and HUT-102 cells, which contain wt proviruses and produce viral particles [39]. To facilitate the observation of Tax-positive cells, C8166 and HUT-102 cells were first mixed with uninfected CEM T cells, giving therefore the background signal. Strikingly, while we used the same procedure that allows easy detection of nuclear bodies in transfected T cells, Tax nuclear bodies were found in less than 8% of the two HTLV-1-infected T cells (Figure5). Importantly, NEMO-enriched perinuclear spots were clearly visible in both C8166 and HUT-102 cells (Figure5, arrow heads), indicating IKK relocalization by endogenous Tax. Moreover, high luciferase production was detected upon transfection of the pNF-κB-luciferase reporter plasmid in these cells (data not shown). 

These results show that Tax, endogenously produced in HTLV-1-infected T cells, does not form nuclear bodies, although it is fully able to activate the NF-κB pathway.

### Tax-induced NF-κB promoter activation correlates with the level of Tax ubiquitination but not the level of Tax SUMOylation

**Figure 6:**
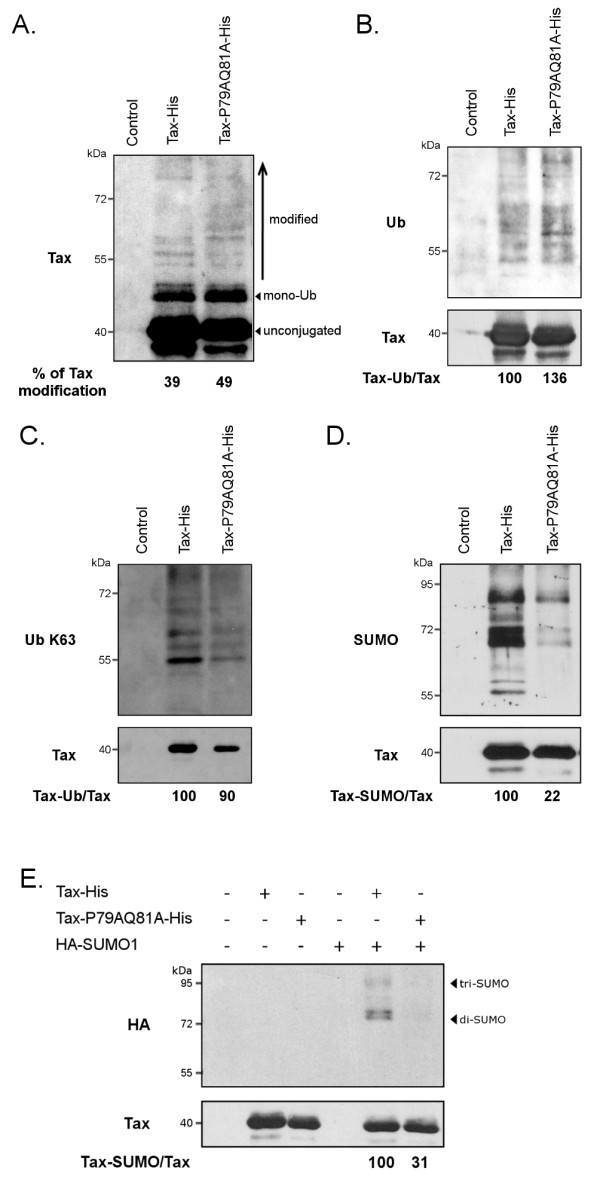
**Tax-P79AQ81A is defective for SUMOylation.** NiNTA experiments performed in HeLa cells showing the total level of Tax modifications (**A**), or the amounts of Tax products conjugated to endogenous total ubiquitin (**B**), endogenous K63-linked ubiquitin chains (**C**), endogenous SUMO (**D**) or overexpressed HA-SUMO-1 (**E**) for either wt Tax and Tax-P79AQ81A. (**A**-**D**) HeLa cells were transfected with a control plasmid or each of the Tax-His constructs and in (**E**) along with a HA-SUMO-1 construct. Tax proteins purified using nickel columns were revealed with anti-Tax sera or with anti-Ubiquitin, anti-K63-linked ubiquitin chains, anti-SUMO-2/3 or anti-HA antibodies, as indicated. The percentage of Tax modification was calculated by dividing the amount of high molecular weight Tax products by the amount of total Tax (modified + unconjugated). The percentages of ubiquitinated or SUMOylated Tax were normalized on the amount of unconjugated Tax (Tax-Ub/Tax) and expressed in comparison to wt Tax (100%).

Since Tax nuclear bodies were previously linked to Tax SUMOylation, we analyzed the post-translational modifications of Tax-P79AQ81A. Purification of wt Tax and the mutant was performed in a highly denaturant guanidine-containing buffer in order to avoid co-purification of non-covalently bound partners. Blotting the purified proteins with a pool of sera from HTLV-1-infected individuals revealed comparable amounts of modified Tax products between wt Tax and Tax-P79AQ81A (39% and 49% of total Tax respectively, Figure6A).

The conjugation of wt Tax and Tax-P79AQ81A to either endogenous ubiquitin or SUMO was next examined. Wt Tax and Tax-P79AQ81A were conjugated to endogenous ubiquitin (Figure6B) and more importantly to endogenous K63-linked ubiquitin chains (Figure6C) at similar levels. Considering that K63-linked ubiquitin chains were shown to be critical for Tax interaction with NEMO, these results are consistent with our data showing that Tax-P79AQ81A binds to NEMO like wt Tax. Contrasting with the level of ubiquitination, Tax-P79AQ81A displayed a severe reduction (78%) in conjugation to endogenous SUMO compared to wt Tax (Figure6D). This reduction of SUMOylation was confirmed by experiments in which Tax or Tax-P79AQ81A was expressed together with a HA-SUMO-1 construct (Figure6E). In all the pulldown experiments, wt Tax and Tax-P79AQ81A were expressed and purified at similar levels (Figure6A-E, Tax panel). Hence, Tax-P79AQ81A is properly ubiquitinated, in particular with K63-linked ubiquitin chains, but is barely SUMOylated. Nevertheless, this mutant is fully able to transactivate a NF-κB promoter, suggesting that SUMOylation is not essential for Tax-induced NF-κB activation.

**Figure 7:**
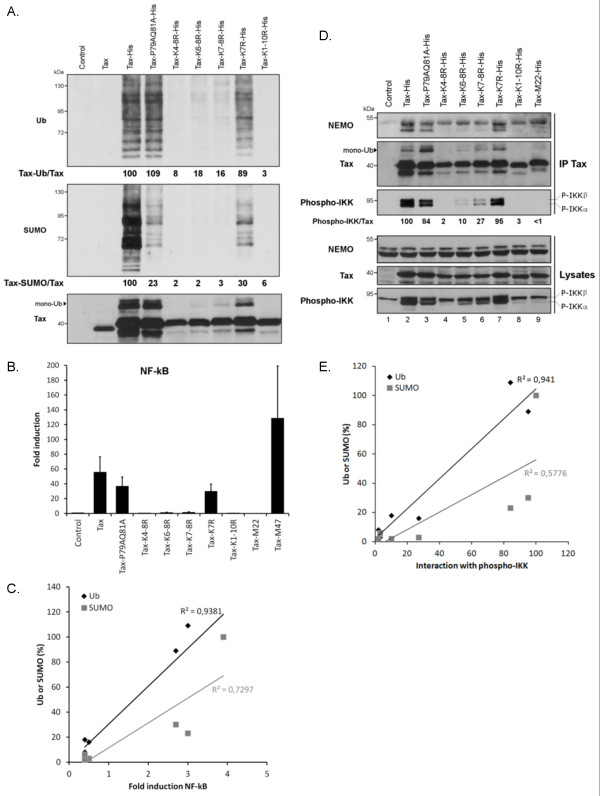
**The degree of Tax NF-**κ**B activity correlates with the levels of Tax ubiquitination but not SUMOylation.** (**A**) NiNTA experiments performed in 293 T cells showing the levels of Tax conjugation to endogenous ubiquitin or SUMO. 293 T cells were transfected as indicated and proteins purified on Nickel columns were revealed using anti-Ubiquitin, anti-SUMO-2/3 or the anti-Tax sera. The percentages of ubiquitinated or SUMOylated Tax were normalized on the amount of unconjugated Tax and expressed in comparison to wt Tax (100%). (**B**) NF-κB reporter assay in 293 T cells. Cells were transfected with control or Tax-His constructs along with the NF-κB or the Renilla luciferase plasmid for normalization. Fold induction was calculated by dividing the firefly/renilla ratio of each Tax protein with the ratio of the control plasmid. Results represent the means and standard error of the means (SEM) from three independent experiments performed in duplicates. (**C**) The level of either ubiquitination or SUMOylation of the Tax mutants was plotted against their NF-κB activities. The regression line shows a correlation between Tax NF-κB activity and ubiquitination (determination coefficient = 0.94) but not SUMOylation (determination coefficient = 0.73). (**D**) Association of the Tax proteins to the IKK complex in 293 T cells. Total proteins (Lysates) or proteins precipitated using the anti-Tax mab (IP Tax) were blotted with anti-NEMO, anti-phospho-IKK or the anti-Tax sera, as indicated. (**E**) The regression line shows a correlation between Tax binding to phospho-IKKα/β and ubiquitination (determination coefficient = 0.94) but not SUMOylation (determination coefficient = 0.58).

To further analyze the relationship between Tax SUMOylation and NF-κB promoter activation, the properties of Tax-P79AQ81A were compared to those of the previously described lysine Tax mutants [24]. NiNTA pulldowns were performed in 293 T cells, in which protein expression is higher than in HeLa cells, ensuring fine quantification. Similarly to what we found in HeLa cells, Tax-P79AQ81A was properly ubiquitinated but barely SUMOylated in 293 T cells (Figure7A). The lysine Tax mutants displayed the previously identified phenotypes: defect in both ubiquitination and SUMOylation (Tax-K1-10R, Tax-K4-8R), reduced ubiquitination and lack of SUMOylation (Tax-K6-8R, Tax-K7-8R) and low SUMOylation with only a slight reduction in ubiquitination (K7R) (Figure7A). Interestingly, this latter pattern is very similar to that of Tax-P79AQ81A. In agreement with our previous studies [24], strong impact of lysine mutations on NF-κB activity of Tax was observed (Figure7B, upper panel). Tax-K6-8R and Tax-K7-8R showed low NF-κB activity (Figure7B) but wt level of CREB promoter activation (Additional file 2: Figure S2E), confirming their specific defect in NF-κB activation. NF-κB reporter gene assays further showed only a slight reduction in the NF-κB activity of Tax-K7R (Figure7B), which mirrored its slight reduction in ubiquitination (Figure7A). Furthermore, when the levels of modifications for each mutant were plotted against the level of NF-κB activities, a linear relation was clearly observed between Tax-induced NF-κB promoter activation and Tax ubiquitination (R^2^ = 0.94) but not Tax SUMOylation (R^2^ = 0.73) (Figure7C). 

Tax ubiquitination is believed to govern Tax binding to NEMO and thereby, IKK activation. Indeed, non-conjugable lysine Tax mutants fail to bind to NEMO [24], and the same defect is observed upon silencing of Ubc13, the ubiquitin conjugating enzyme shown to mediate Tax conjugation to K63-linked ubiquitin chains [30]. We further assessed the role of Tax ubiquitination on NEMO binding by analyzing the ability of the mutants used above to bind to either endogenous NEMO or endogenous phospho-IKKα/β (Figure7D). Like for NF-κB promoter activation, a linear relation was found between the amount of phospho-IKK associated to Tax and Tax ubiquitination (R^2^ = 0,94) but not SUMOylation (R^2^ = 0,58) (Figure7E). These results provide direct evidence that ubiquitinated Tax is the species that binds to IKK and triggers IKK activation.

Altogether these data demonstrate that low SUMOylation does not prevent Tax-induced NF-κB activation and that Tax ubiquitination is the predominant determinant for Tax-induced NF-κB activation.

### Fusion of SUMO-1 increases the ubiquitination of Tax-P79AQ81A

**Figure 8:**
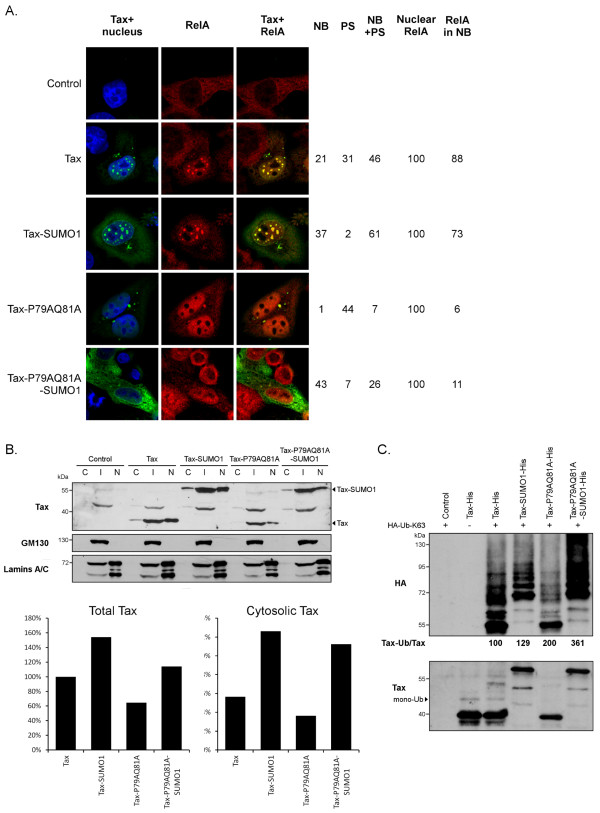
**SUMO-1 fusion increases the cytosolic level and ubiquitination of Tax-P79AQ81A.** (**A**) Confocal microscopy analysis showing the distribution of Tax or Tax-P79AQ81A fused or not to SUMO-1 (green) and of endogenous RelA (red) as well as nucleus staining (blue). The percentages of cells containing Tax in only nuclear bodies (NB), perinuclear spots (PS) or in both locations (NB + PS) are indicated. The percentages of cells containing RelA in the nucleus and the percentages of nuclear bodies-positive cells containing RelA in nuclear bodies are also indicated. At least 100 cells were analyzed in each condition. (**B**) Cell fractionation analysis. Cell extracts were separated in a cytosolic (**C**), intermediate (I) and nuclear (N) fraction as described [32]. Upper panel: Proteins were blotted with either an anti-Tax sera; anti-GM130, a marker of the intermediate fraction (Golgi apparatus), or anti-Lamins A/C, a marker of the nuclear fraction. Lower panel: the intensity of the Tax bands in all three fractions was quantified (Total Tax) and normalized to 100% for wt Tax. The proportion of Tax in the cytosolic fraction was calculated by dividing the intensity of each cytosolic Tax band by the value of Total Tax. (**C**) Ubiquitination of non-fused or fused wt Tax and Tax-P79AQ81A proteins. Ni-NTA experiments were performed in HeLa cells transfected with a control plasmid or the Tax-His constructs together with a HA-Ub-K63 plasmid. The percentages of HA-Ub-K63 conjugated products were normalized on the amount of unconjugated Tax and expressed in comparison to wt Tax (100%).

We previously reported that fusing SUMO-1 to certain lysine Tax mutants partially rescued their ability to form nuclear bodies [24]. We thus wondered whether fusion of SUMO-1 to Tax-P79AQ81A could restore its nuclear body localization. Fusion of SUMO-1 to wt Tax increased the proportion of cells containing Tax nuclear bodies, as previously described [20,24] (98% of total NB (NB and NB + PS) for Tax-SUMO-1 as compared to 67% for non-fused Tax). A higher proportion of cells containing nuclear bodies was also observed upon SUMO-1 fusion to Tax-P79AQ81A (69% for Tax-P79AQ81A-SUMO-1, as compared to 8% for Tax-P79AQ81A). However, these nuclear bodies were less numerous (usually 2 to 3 per nucleus) than in the case of wt Tax and were much smaller than those formed by wt Tax (Figure8A). Moreover, the recruitment of RelA within these small nuclear bodies were only seen in 11% of nuclear bodies-containing cells, showing that fusion of SUMO-1 did not allow the formation of nuclear bodies with wild-type phenotype (Figure8A). Confocal experiments also showed that fusion of SUMO-1 slightly decreased the number of cells containing Tax in perinuclear spots, an effect observed for both wt Tax (63% of Tax-SUMO-1 expressing cells as compared to 77% of wt Tax expressing cells) and Tax-P79AQ81A (33% of Tax-P79AQ81A-SUMO-1 expressing cells as compared to 51% of Tax-P79AQ81A expressing cells). Moreover, an increased cytosolic staining was observed for both wt Tax-SUMO-1 and Tax-P79AQ81A-SUMO-1, as compared to their non-fused counterparts. 

To confirm this, cell fractionation experiments were performed, in which cell extracts were separated in soluble cytosolic, intermediate (containing the perinuclear spots) and nuclear fractions, as previously described [32] (Figure8B, upper panel). Quantification of the amounts of Tax in all three fractions (total, lower panel) revealed that the total amounts of Tax-SUMO-1 or Tax-P79AQ81A-SUMO-1 were increased compared to their respective non-fused counterpart. This increase was due to higher amounts of either Tax-SUMO-1 or Tax-P79AQ81A-SUMO-1 in the cytosolic fraction (lower panel) with few changes in the two other fractions (quantification not shown). This confirms the confocal microscopy observations (Figure8A) and suggests that fusion of SUMO-1 stabilizes Tax in the cytosol.

Since Tax conjugation to K63-linked ubiquitin chains was correlated to the cytoplasmic localization of Tax [32], we compared the levels of ubiquitination of fused and non-fused proteins (Figure8C). Strikingly, we found that fusion of SUMO-1 increased the conjugation to K63-linked ubiquitin chains of Tax-P79AQ81A but not of wt Tax.

Altogether these results indicate that fusion of SUMO-1 only partially rescues the formation of nuclear bodies by Tax-P79AQ81A. Moreover, they reveal an unexpected effect of the SUMO-1 fusion that stabilizes wt Tax and Tax-P79AQ81A in the cytosol and increases the ubiquitination of the mutant.

## Discussion

In this study, we directly analyzed the role of Tax nuclear bodies and Tax SUMOylation on NF-κB activation.

Previous studies have described that Tax forms nuclear spots called Tax nuclear bodies or Tax speckled structures [16,17]. A Tax speckled structure localization signal (TSLS) positioned between residues 50 and 75 was subsequently mapped in the Tax sequence [18]. In order to identify new functional motifs of Tax, we selected the PxQxT motif at position 79–83 because it fits with a TRAF-binding motif and is adjacent to the TSLS. We found that this motif does not control Tax interaction with the TRAF since its mutation alters neither Tax ubiquitination (this study) nor the co-precipitation of Tax with either TRAF2 or TRAF5 (data not shown). In contrast, mutations of the P79 and Q81 residues dramatically reduce the formation of Tax nuclear bodies, confirming the role of the TSLS and showing that this sequence includes the PxQ motif.

We and others have demonstrated that Tax ubiquitination, especially conjugation to K63-linked ubiquitin chains, permits Tax binding to NEMO [24,30,32]. Here we report that Tax-P79AQ81A, which is conjugated to either total ubiquitin or K63-linked ubiquitin chains at the same level than wt Tax, binds to NEMO and activates IKK like wt Tax. Moreover, analysis of a series of Tax mutants allowed us to show that the amount of endogenous phospho-IKKα/β coprecipitated with Tax correlates with the level of Tax ubiquitination but not SUMOylation. These findings confirm the critical role of Tax ubiquitination in NEMO binding and IKK activation and also demonstrate that Tax SUMOylation is dispensable for these processes. Tax conjugation to K63-linked ubiquitin chains was also shown to relocalize Tax and NEMO to perinuclear spots [32,33]. However, recent findings showed that NEMO targeting to perinuclear spots is also impaired upon siRNA-mediated SUMO silencing [19]. Using both microscopy analysis and cell fractionation, we found that endogenous NEMO is recruited in perinuclear spots at the same level in cells producing either wt Tax or Tax-P79AQ81A. Hence, lowering Tax SUMOylation has no effect on NEMO targeting to cytoplasmic spots. The effect of SUMO silencing could be explained by the role of a minor fraction of SUMOylated Tax or of a SUMOylated unknown substrate in the cytoplasmic targeting of NEMO.

Tax nuclear bodies were previously reported to contain RelA and NEMO and therefore identified as transcriptionally active structures [16,19,20]. Surprisingly, we found that lack of nuclear body formation by Tax-P79AQ81A does not prevent this mutant from activating both the cytoplasmic and nuclear steps of the NF-κB pathway. Importantly, such proper NF-κB promoter activation was observed not only in adherent cells but also in T cells, notably CD4^+^ primary T cells. The composition of Tax nuclear bodies has essentially been studied in transfected adherent cells [15-17], and we were indeed able to detect these structures in all transfected cells, including T cells. However, using the same staining procedure, we observed that Tax nuclear bodies are nearly absent in HTLV-1-infected T cells. Hence, Tax nuclear bodies appear to be visible when Tax is transiently produced but not in an endogenous situation. This may suggest that nuclear bodies represent a storage compartment rather than transcriptionally active structures. However, it cannot be excluded that small clusters of Tax, undetectable by confocal microscopy, are indeed formed in HTLV-1-infected T cells. Further investigations are therefore needed to clarify the pattern and role of nuclear Tax, in particular in HTLV-1-infected CD4^+^ T cells.

Our work also allowed us to further explore the relationship between Tax ubiquitination and Tax SUMOylation. Indeed, as mentioned above, Tax-P79AQ81A mutant whose endogenous SUMOylation is reduced by around 80% is ubiquitinated at the same level as wt Tax, both in terms of total ubiquitination and specific conjugation to K63-linked ubiquitin chains. This strongly suggests that Tax SUMOylation is dispensable for Tax ubiquitination. That Tax SUMOylation may represent a signal for Tax ubiquitination was indeed recently proposed based on findings showing that RNF4, a SUMO-targeted ubiquitin ligase (STUbL), was able to modulate Tax ubiquitination [27]. It was shown that RNF4 induced the ubiquitination of a SUMO-1 fused recombinant Tax protein *in vitro* and that siRNA-mediated depletion of RNF4 abolished Tax ubiquitination. However, we found here that the SUMO-1 fused form of Tax was ubiquitinated at comparable level as non-fused Tax in HeLa cells. Moreover, we show that in contrast to RNF4 depletion, low Tax SUMOylation does not prevent Tax ubiquitination in cells. Of note, a GFP-tagged Tax was used in the RNF4 study [27] while our experiments were performed using a Tax-6his construct, which could lead to difference in Tax modifications and/or localization. In addition, it cannot be excluded that the low residual level of SUMOylation of Tax-P79AQ81A could be still sufficient to promote Tax ubiquitination. However, this would likely have been associated to a certain degree of reduction of Tax ubiquitination, as observed in RNF4-depleted cells [40]. Along with these findings, our data suggest therefore that RNF4 may not directly modulate wild-type Tax ubiquitination, but acts in an indirect manner by interfering with ubiquitination machineries or with direct regulators of Tax ubiquitination.

We previously concluded that ubiquitination and SUMOylation were both required for optimal NF-κB activation by Tax through analysis of lysine mutants and SUMO-1-fused proteins. In this study, we revisited the role of Tax SUMOylation through a direct approach based on an ubiquitinated but intrinsically weakly SUMOylated Tax mutant. We found that Tax-P79AQ81A retains most of the NF-κB activity of wt Tax, while in the same conditions, very little NF-κB activity was measured for a mutant defective for both ubiquitination and SUMOylation (Tax-K4-8R). Potent NF-κB activation by Tax-P79AQ81A was not only observed in 293 T cells and HeLa cells but also in T cells. Indeed, Tax-P79AQ81A is as active as wt Tax in CEM T cells and more importantly, in primary CD4^+^ T lymphocytes. Of note, study of another mutant, K7R, confirms that weak SUMOylation does not preclude NF-κB activation. Finally, we were able to document that the abilities to activate a NF-κB promoter of a series of Tax mutants correlate with their levels of ubiquitination but not of SUMOylation. It could be argue that low level of SUMOylation would be sufficient to regulate some Tax activities, even in absence of Tax nuclear bodies. However, our findings provide strong evidence that Tax SUMOylation is not a key determinant for Tax-induced NF-κB activation.

That low SUMOylation does not alter Tax-induced NF-κB activation appears to contradict our previous findings showing that fusion of SUMO-1 to lysine mutants restored their NF-κB activities. However, we believe that our current and earlier data can be reconciled in light of recent findings from other groups. Indeed, we and others previously noticed that the SUMO-1 fusion restores the NF-κB activity of some but not all lysine Tax mutants: i.e., it restores the NF-κB activities of Tax-K6-8R or Tax-K7-8R (also referred to as Tax-R4-6 K), which retains partial ubiquitination, but not that of Tax-K4-8R, which is no longer ubiquitinated [20,24]. Moreover, we documented that fusion of SUMO-1 does not increase the NF-κB activity of either wt Tax [24] or Tax-P79AQ81A (data not shown), which are both fully ubiquitinated. Fusion of SUMO-1 appears, therefore, to only enhance the NF-κB activity of partially but not fully ubiquitinated Tax proteins. As mentioned above, RNF4 was recently shown to induce the ubiquitination of a recombinant GFP-Tax protein fused to SUMO-1 *in vitro*[27]. Hence, artificial fusion of SUMO-1 to the Tax mutants may similarly enhance their ubiquitination by facilitating the interaction with RNF4. Strikingly, it was previously reported that fusion of SUMO-1 increases the endogenous ubiquitination of Tax-K7-8R/Tax-R4-6 K but not Tax-K4-8R [20]. Furthermore, we show here that fusion of SUMO-1 stabilizes both Tax and Tax-P79AQ81A in the cytosol but only significantly increases ubiquitination of Tax-P79AQ81A. This latter effect is reminiscent of the cytoplasmic relocalization and enhanced ubiquitination of the GFP-tagged Tax construct upon RNF4 overexpression [27]. Why RNF4 would only increase the ubiquitination of Tax mutants but not wt Tax remains to be elucidated. Because these proteins differ by the presence or absence of SUMOylation, it could be speculated that the natural SUMO chains of Tax may somehow prevent the interaction of wt Tax or Tax-SUMO-1 with RNF4. Whatever the exact mechanism involved, these findings support the view that the effect previously attributed to the fusion of SUMO-1 to lysine Tax mutants was actually linked to Tax stabilization and/or facilitation of Tax ubiquitination.

## Conclusions

Our findings provide strong evidence that Tax SUMOylation and formation of Tax nuclear bodies are dispensable for proper NF-κB pathway activation by Tax, especially in natural target cells of HTLV-1 infection. In contrast, preventing both ubiquitination and SUMOylation of Tax is detrimental for NF-κB activation, highlighting the critical importance of the ubiquitin-dependent cytoplasmic events that involve the Tax/IKK interaction. Targeting ubiquitination pathways has emerged as a new promising therapeutic approach of malignancies [41]. Given the critical role that Tax-induced NF-κB activation plays in HTLV-1-induced T cell transformation [5], such approach would be especially relevant in the treatment of HTLV-1-induced T-cell malignancies.

## Methods

### Cell culture and transfection

HeLa and 293 T cells were grown in Dulbecco’s modified Eagle’s medium supplemented with 10% fetal calf serum, 2 mM glutamine and antibiotics (Invitrogen) and were transfected using the lipofectamine reagent (Invitrogen). HTLV-1-infected T cells C8166 and HUT-102 as well as HTLV-1-negative CEM T cells were grown in RPMI 1640 medium supplemented as above along with 0.5% glucose and were transfected using the DMRIE-C reagent (Invitrogen), following the manufacturer’s instructions.

Primary human CD4^+^ T cells were purified from peripheral blood samples of healthy donors from EFS (Etablissement Français du Sang, Paris). After separation of mononuclear cells by density gradient, CD4+ T cells were isolated by positive selection using CD4^+^ T lymphocyte enrichment immunomagnetic beads (Beckton Dickinson, France). Purified CD4^+^ T cells were then cultured in RPMI 1640 medium supplemented as above and containing 10% inactivated human serum (Sigma Aldrich) along with 50 IU/mL interleukin-2 (IL-2, PeproTech, France) and 3 mg of phytohemaglutinin-M (PHA-M, Sigma). CD4^+^ T cells were transfected using the Amaxa nucleofector (VPA-1002, Lonza) with the T23 program following the manufacturer’s instructions.

### Plasmids

All the Tax constructs used in this study encode proteins fused to a C-terminal 6His tag (His). Tax-His, lysine Tax mutants, Tax-M22-His and Tax-M47-His cloned in the pSG5M vector, HTLV-1-LTR-Luc, NF-κB-Luc and pRL-TK plasmids were described elsewhere [24,32]. To generate the Tax-P79AQ81A mutant, a fragment comprised between the EcoRI and PmlI restriction sites of the Tax cDNA was mutated and amplified using the following primers: forward mutagenic Tax primer [5’CTCCTTCGCGACCGCGAGAACCTCTAAG3’] and reverse mutagenic Tax primer [5’CTTAGAGGTTCTCGCGGTCGCGAAGGAG3’] as well as the cloning primers TaxEcoF [5’GTAATACGACTCACTATAGGGCGAATTC3’] and TaxPmlIR [5’CACGTGGGGCAGGAGGGGCCAGGTG3’]. Two separated PCR reactions were first performed using either the forward mutagenic Tax primer and TaxPmlIR or the reverse mutagenic Tax primer and TaxEcoF, and the two amplified fragments were mixed and used to perform a global PCR reaction using the TaxEcoF and TaxPmlIR primers. The final PCR product was then cloned in the pTOPO vector (Invitrogen), and the EcoRI-PmlI fragment of pTOPO-Tax-P79AQ81A was finally introduced into the pSG5M vector. Correct introduction of the mutation was attested by the presence of a NruI site (introduced into the mutagenic primers). The Tax-P79AQ81A mutation was then introduced in the Tax-SUMO-1-6His plasmid by exchanging the EcoRI-PmlI fragments. The integrity of all constructs was verified by sequencing.

### Antibodies

Tax was detected using a pool of sera from HTLV-1 infected individuals (anti-Tax sera) or the anti-Tax monoclonal antibody (mab) 168-A51 (NIH AIDS Research and Reference Reagent Program, USA). The following primary antibodies were used: Ubiquitin (P4D1 from Tebu Bio or Fk2 from Millipore), Ub-K63 chains (D7A11; Cell Signaling Technology), SUMO 2/3 (ab3742; Abcam), HA-tag (12CA5; Roche), RelA/p65 (sc-372) and IKKγ (sc-8330)(Santa Cruz) phospho-IKKα/β (C84E11; Cell Signaling), GM-130 (610822, BD Biosciences) and Lamin A/C (2032, Cell signaling). HRP-conjugated anti-human, anti-mouse and anti-rabbit IgG (Promega) were used as secondary antibodies in western blot. The following secondary antibodies were used for immunostaining: goat anti-rabbit IgG conjugated to Alexa Fluor 594 or Cyanine 5 (Invitrogen) and donkey anti-mouse IgG conjugated to fluorescein isothiocyanate (FITC; Jackson Immunoresearch).

### Luciferase assays

Luciferase assays were performed in duplicates in 24-well plates for HeLa or 293 T cells (3 × 10^4^/well), CEM T cells (4 × 10^5^/well) or sorted CD4^+^ primary T cells (2.5 × 10^6^/well). CD4+ primary T cells were starved by removing PHA and IL-2 from the culture medium 24 h before transfection. HeL, 293 T and CEM cells were cotransfected with 500 ng of either the NF-κB-Luc or HTLV-1-LTR-Luc (CREB) reporter plasmid, 50 ng of the Renilla reporter plasmid pRL-TK and 1 μg of the control or the Tax plasmids. CD4^+^ primary T cells were cotransfected with 2 μg of either the NF-κB-Luc or HTLV-1-LTR-Luc reporter plasmid, 500 ng of the Renilla reporter plasmid pRL-TK and 3 μg of the control or the Tax plasmids. Luciferase activity was quantified 24 h post-transfection for 293 T cells or 48 h post-transfection for HeLa cells or T cells using the Dual Luciferase Assay System (Promega) and values were normalized with Renilla activity. Statistical analyses were performed using the student test.

### Cell lysis, cell fractionation, immunoprecipitation and immunoblot

Preparations of cellular aggresome were performed as described in [37]. Cell fractionation in cytosolic, intermediate and nuclear fractions was peformed as described in [32].

Immunoprecipitations were carried out as follow: at 24 h post-transfection, HeLa cells were lysed in lysis buffer (50 mM Tris–HCl pH8, 1% NP40, 0.5% deoxycholate, 0.1% SDS and 150 mM NaCl) supplemented with protease and phosphatase inhibitors (Roche). Cell lysates were incubated overnight with the anti-Tax mab at 4°C, and antibody complexes were captured on protein G-sepharose beads (GE Healthcare) 1 h at 4°C. Sepharose beads were then washed 5 times in wash buffer (120 mM NaCl, 20 mM Tris–HCl pH8, 0.2 mM NaF, 0.2 mM EGTA, 0.2% deoxycholate, 0.5% NP40) before elution in Laemmli buffer.

Proteins in cell fractions, immunoprecipitated proteins and total cell lysates were separated by SDS-PAGE, transferred to nitrocellulose membranes and blotted with specific antibodies. For quantification, shortly exposed films were scanned with an AGFA scanner, and signal densities of proteins were measured with ImageJ software (Wayne Rasband; NIH). Signal density in an empty lane was also measured and subtracted from the signal of each band.

### Ni-NTA pull-down

At 24 h post-transfection, HeLa cells were lysed in reducing and highly denaturing conditions in buffer A (6M Guanidium-HCl, 0.1M NaH_2_PO_4_, 10 mM imidazole, pH 8) and incubated with Ni^2+^-NTA (nitrilotriacetic acid) beads (Sigma) for 3 h at room temperature. The beads were washed three times in buffer A, twice in buffer B (buffer A diluted 1:4 in buffer C) and twice in buffer C (25 mM Tris–HCl pH 6.8, 10 mM imidazole). The proteins bound were eluted in Laemmli buffer, separated by SDS-PAGE, transferred to nitrocellulose membrane and blotted with specific antibodies. Film quantification was performed as described above.

### Confocal analysis

HeLa or 293 T cells were seeded on glass coverslips in 24-well plates the day before transfection and staining was performed 24 h post-tranfection. At 24 h post-transfection, transfected CEM T cells were washed once with PBS and deposited onto poly-L-lysine coated coverslips in RPMI 1640 medium for 1 h at 37°C. Both cell types were washed twice with PBS, fixed with 4% paraformaldehyde for 15 min, washed twice with PBS, permeabilized with cold methanol for 5 min, and washed twice with PBS. The cells were incubated in PBS containing 2% BSA and 0.1% Tween for 30 min and then with primary antibodies diluted in PBS buffer containing 2% BSA and 0.1% Tween for 1 h. After 3 washes with PBS containing 0.1% Tween, the cells were incubated with secondary antibodies diluted in PBS buffer containing 2% BSA and 0.1% Tween for 45 min. The cells were washed with PBS-Tween buffer, and incubated with DAPI (Sigma) diluted in PBS for 10 min for nucleus staining. After two washes with PBS, the coverslips were mounted in FluorSave Reagent (Calbiochem). Laser scanning microscopy was performed using a Leica TCS resonant scanner multi-photon (spinning disc) with a 63X objective, and images were analyzed using ImageJ software.

## Competing interests

The authors declare that they have no competing interests.

## Authors’ contributions

A.Bo. designed and performed the work, analyzed the data and wrote the paper. V.R. performed experiments with primary cells, analyzed the data and edited the paper. P.N and M.N contributed to biochemical experiments and analyzed data. M.C., L.W. and S.P. contributed to molecular biology experiments. A.Ba. and RM contributed reagents and ideas for the work and edited the paper. L.B. designed experiments and edited the paper. CP designed and supervised the study and wrote the paper. All authors approved the submitted manuscript.

## Supplementary Material

Additional file 1 ** Figure S1: Subcellular localization of wt Tax and Tax-P79AQ81A in the cells.** Description of data. Confocal microscopy analysis performed in HeLa cells showing Tax localization (green). Nuclei were stained with DAPI (blue). The percentages of cells containing Tax in only nuclear bodies (NB), in only perinuclear spots (PS) or in nuclear bodies + perinuclear spots (NB + PS) are indicated for both wt Tax and Tax-P79AQ81A. At least 200 cells were analyzed. NB and PS are indicated by arrows and arrow heads, respectively. Click here for file

Additional file 2 ** Figure S2. CREB promoter activities of the Tax proteins used in the study.** Description of data. (A-D) Comparison of the CREB promoter activities of wt Tax and Tax-P79AQ81A in Tax-transfected HeLa cells (A), 293 T cells (B), CEM T cells (C) and primary CD4^+^ T cells (D). Cells were transfected with a control plasmid or with the Tax-His constructs along with the CREB reporter plasmid and the Renilla luciferase expression plasmid for normalization. (E) CREB promoter activities of the lysine Tax mutants in 293T cells. In all experiments, the M22 (defective for the NF-κB pathway) and M47 (defective for the CREB pathway) mutants were included as controls. Fold induction was calculated by dividing the firefly/renilla ratio of each Tax protein with the firefly/renilla ratio obtained with the control plasmid. The results represent the means and standard error of the means (SEM) from at least four independent experiments performed in duplicates. Click here for file
