# Supplementary figures and images for: Low nuclear body formation and tax SUMOylation do not prevent NF-kappaB promoter activation
Source: Retrovirology. 2012 Sep 25;9:77. doi: 10.1186/1742-4690-9-77 (PMC3476979; doi:10.1186/1742-4690-9-77)

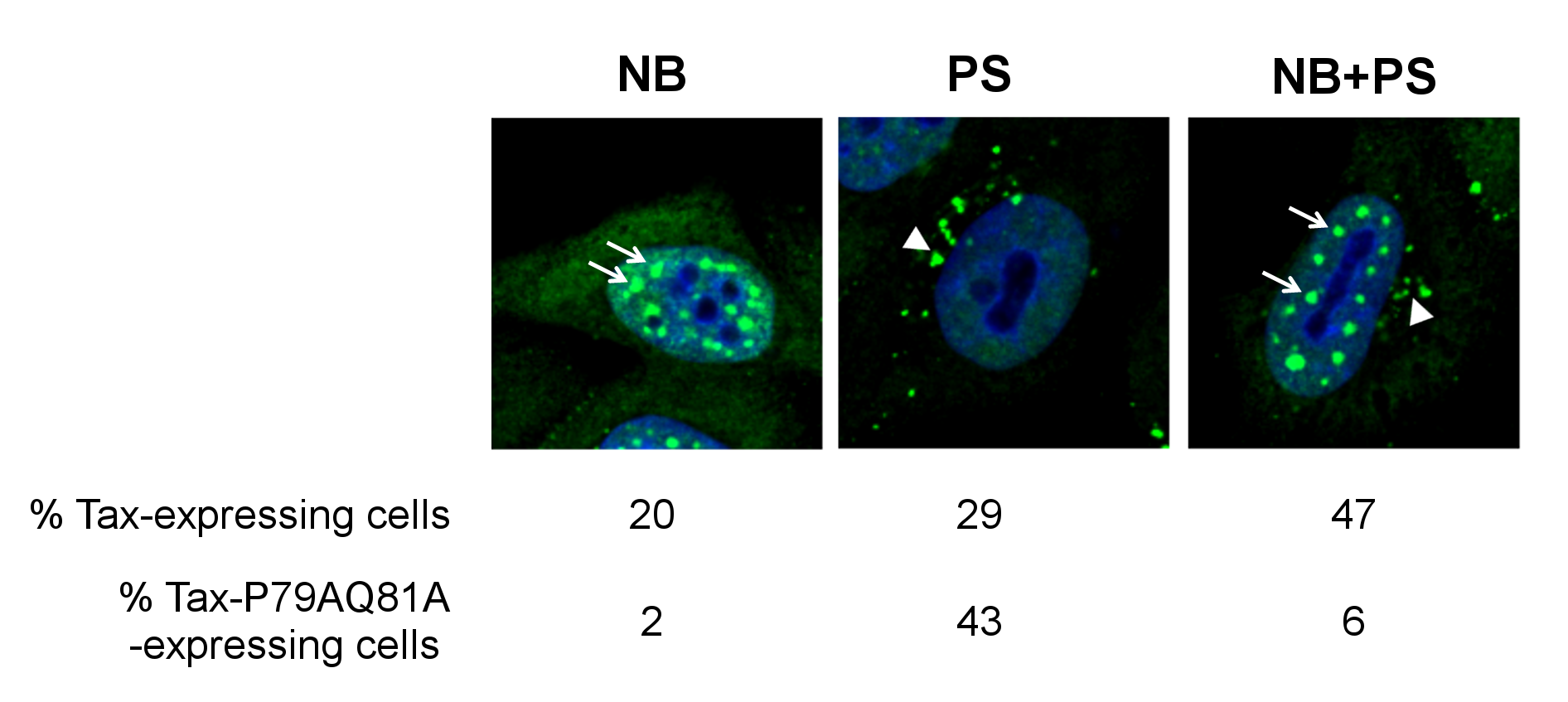

Supplement: Additional file 1 — Figure S1: Subcellular localization of wt Tax and Tax-P79AQ81A in the cells. Description of data. Confocal microscopy analysis performed in HeLa cells showing Tax localization (green). Nuclei were stained with DAPI (blue). The percentages of cells containing Tax in only nuclear bodies (NB), in only perinuclear spots (PS) or in nuclear bodies + perinuclear spots (NB + PS) are indicated for both wt Tax and Tax-P79AQ81A. At least 200 cells were analyzed. NB and PS are indicated by arrows and arrow heads, respectively. [file 1742-4690-9-77-S1.tiff]

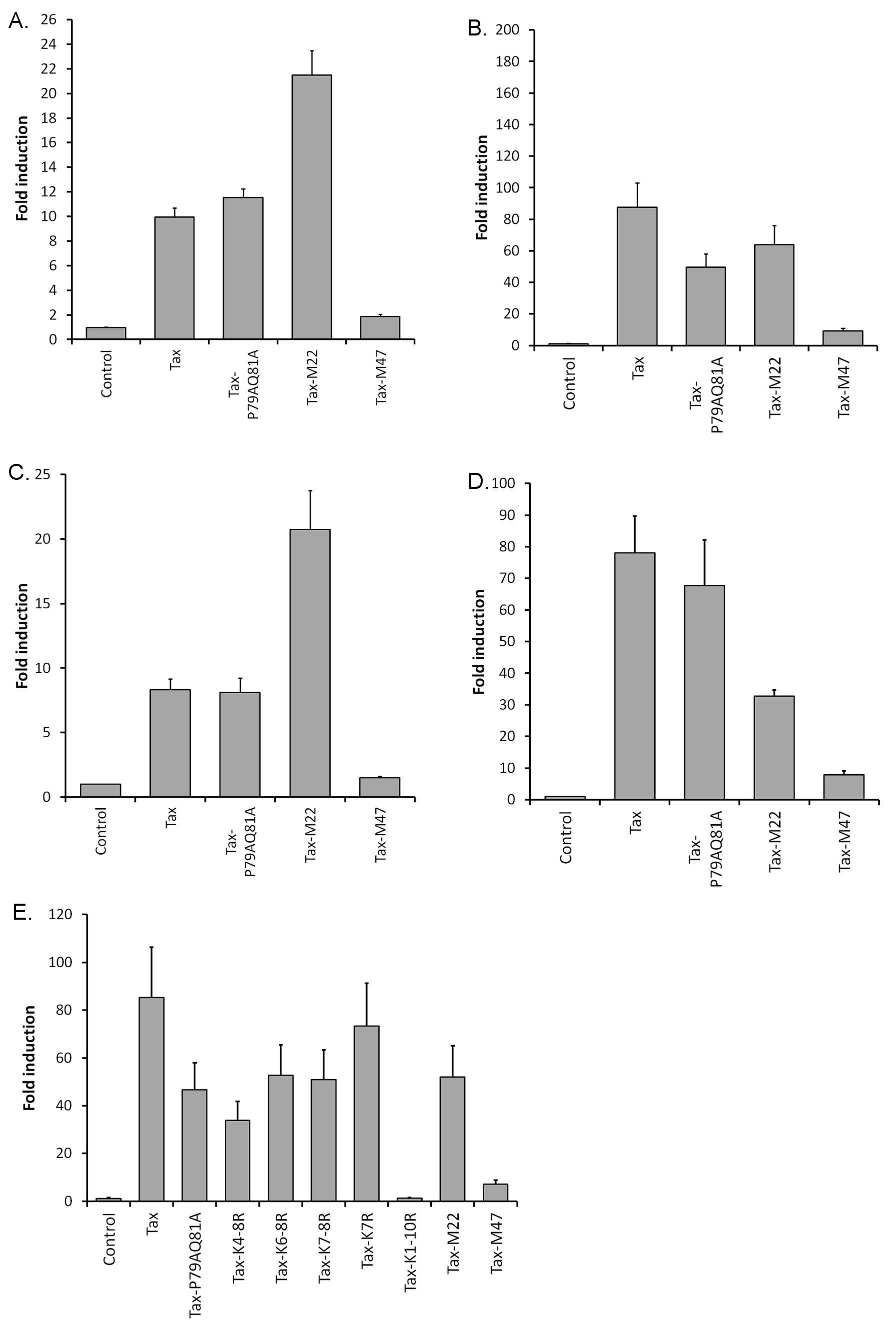

Supplement: Additional file 2 — Figure S2. CREB promoter activities of the Tax proteins used in the study. Description of data. (A-D) Comparison of the CREB promoter activities of wt Tax and Tax-P79AQ81A in Tax-transfected HeLa cells (A), 293 T cells (B), CEM T cells (C) and primary CD4+ T cells (D). Cells were transfected with a control plasmid or with the Tax-His constructs along with the CREB reporter plasmid and the Renilla luciferase expression plasmid for normalization. (E) CREB promoter activities of the lysine Tax mutants in 293T cells. In all experiments, the M22 (defective for the NF-κB pathway) and M47 (defective for the CREB pathway) mutants were included as controls. Fold induction was calculated by dividing the firefly/renilla ratio of each Tax protein with the firefly/renilla ratio obtained with the control plasmid. The results represent the means and standard error of the means (SEM) from at least four independent experiments performed in duplicates. [file 1742-4690-9-77-S2.tiff]
